# Knockout Mouse Studies Show That Mitochondrial CLPP Peptidase and CLPX Unfoldase Act in Matrix Condensates near IMM, as Fast Stress Response in Protein Assemblies for Transcript Processing, Translation, and Heme Production

**DOI:** 10.3390/genes15060694

**Published:** 2024-05-27

**Authors:** Jana Key, Suzana Gispert, Georg Auburger

**Affiliations:** Experimental Neurology, Clinic of Neurology, University Hospital, Goethe University Frankfurt, Heinrich Hoffmann Str. 7, 60590 Frankfurt am Main, Germany; jana.key@unimedizin-ffm.de (J.K.); gispert-sanchez@em.uni-frankfurt.de (S.G.)

**Keywords:** Perrault syndrome type 3 (PRLTS3), iron toxicity, pyridoxal-5′-phosphate, VWA8, GFM1, PNPT1, RNA-G4, ISC, ALAS, OAT

## Abstract

LONP1 is the principal AAA+ unfoldase and bulk protease in the mitochondrial matrix, so its deletion causes embryonic lethality. The AAA+ unfoldase CLPX and the peptidase CLPP also act in the matrix, especially during stress periods, but their substrates are poorly defined. Mammalian CLPP deletion triggers infertility, deafness, growth retardation, and cGAS-STING-activated cytosolic innate immunity. CLPX mutations impair heme biosynthesis and heavy metal homeostasis. CLPP and CLPX are conserved from bacteria to humans, despite their secondary role in proteolysis. Based on recent proteomic–metabolomic evidence from knockout mice and patient cells, we propose that CLPP acts on phase-separated ribonucleoprotein granules and CLPX on multi-enzyme condensates as first-aid systems near the inner mitochondrial membrane. Trimming within assemblies, CLPP rescues stalled processes in mitoribosomes, mitochondrial RNA granules and nucleoids, and the D-foci-mediated degradation of toxic double-stranded mtRNA/mtDNA. Unfolding multi-enzyme condensates, CLPX maximizes PLP-dependent delta-transamination and rescues malformed nascent peptides. Overall, their actions occur in granules with multivalent or hydrophobic interactions, separated from the aqueous phase. Thus, the role of CLPXP in the matrix is compartment-selective, as other mitochondrial peptidases: MPPs at precursor import pores, m-AAA and i-AAA at either IMM face, PARL within the IMM, and OMA1/HTRA2 in the intermembrane space.

## 1. CLPP Is a Key Modifier of Growth and Lifespan, but Its Substrates Remain Unclear

LONP1 is the principal proteolysis factor of the mitochondrial matrix, combining an AAA+ ATPase unfoldase domain with a serine peptidase domain within the same protein sequence. LONP1 homo-multimerizes in a ring or barrel shape to maximize its efficiency and plays a crucial role in the turnover of respiratory chain complexes and most other proteins in this compartment [1,2,3]. According to studies on bacteria, CLPXP is perceived as a similar but stress-responsive proteolysis machine, also in the mitochondrial matrix. However, CLPX as an AAA+ ATPase unfoldase component and CLPP as a serine peptidase component are separate proteins. To obtain proteolytic capacity, via assembly in a barrel-like conformation similar to LONP1, they can hetero-multimerize. Nonetheless, in proteolysis, they play a secondary role, becoming prominent only after cellular stress [4]. Both systems have been conserved from bacteria to humans, so each of them has to play very relevant roles in the mitochondrial matrix. Indeed, the loss of LONP1 in a homozygous state causes lethality already during early embryonic development [1]. In contrast, the loss of CLPP was observed to extend the lifespan in the eukaryotic fungus *Podospora anserina*, and CLPP is constitutively absent from the yeast *Saccharomyces cerevisiae* [5,6]. This emphasizes a dramatic difference in the functional impact of these two systems, which is at odds with the idea that both act similarly in proteolytic degradation. 

Genetic analyses of human diseases recently showed mild LONP1 mutations to be responsible for CODAS syndrome, where craniofacial dysmorphia, cataracts, ptosis, a median nasal groove, delayed tooth eruption, delayed epiphyseal ossification, metaphyseal hip dysplasia, vertebral coronal clefts, short stature, psychomotor and developmental delay, and hearing loss are diagnostic hallmarks [7,8]. In contrast, CLPP mutations cause Perrault syndrome type 3 (PRLTS3) [9,10,11,12,13,14,15]. Perrault syndrome was clinically and genetically defined as the combination of early female infertility due to primary ovarian failure, with the subsequent onset of progressive sensorineural hearing impairment and autosomal recessive inheritance. Later, it was observed that not only deafness but also sensory neuropathy, ataxia, and brain white matter changes can appear as neurodegenerative features [16,17,18,19,20]. Judging by human genetics, the functions of LONP1 and CLPP therefore appear to target different pathways, and their dysfunctions have widely different consequences.

Genetic causes of PRTLS are almost exclusively due to mtDNA/mtRNA or mitoribosome machinery errors [13,21]. The role of mtDNA in Perrault syndrome’s pathogenesis is substantiated by causal mutations in the mitochondrial DNA/RNA helicase TWNK/PEO1 and in the mitochondrial transcription factor TFAM [22,23]. The role of mtRNA processing is corroborated by causal mutations in the mitochondrial rRNA chaperone ERAL1 and the mitochondrial RNase P component PRORP [16,24,25]. The roles of mt-tRNA processing and mitoribosomal translation are evident from causal mutations in the mitochondrial tRNA–amino acid ligases HARS2 and LARS2, as well as the mitoribosome-associated factor RMND1 [13,26,27,28,29,30,31,32,33,34,35]. The detailed correlation of mutant mitochondrial factors with the consequent phenotypes (Figure 4 in [36]) indicates that primary infertility is mostly due to abnormal mtDNA or mt-tRNA processing, whereas hearing impairment is frequently due to abnormal mtRNA processing or mitoribosomal translation. Thus, CLPP appears to selectively modulate mitochondrial RNA processing and translation. 

Also in a complete phenotypic contrast, human CLPX mutations were observed to cause erythropoietic protoporphyria 2 (leading to acute skin photosensitivity, mild microcytic anemia, and rarely, severe liver disease) [37,38]. The credibility of these findings is enhanced by observations from yeast to humans that CLPX, independently from CLPP, activates heme biosynthesis [5,37,39].

**Figure 1:**
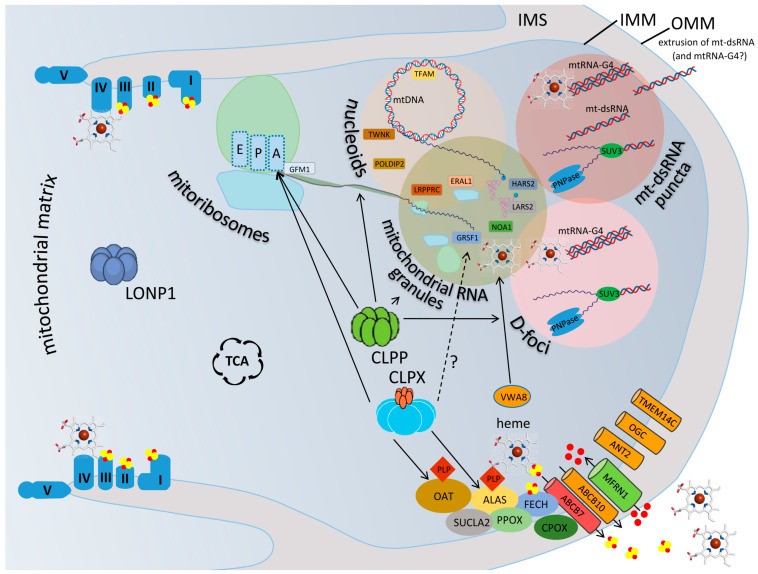
CLPX and CLPP perform first aid in matrix granules where reaction intermediates are separated from the aqueous phase. A depiction of a mitochondrial matrix compartment between two cristae, where the LONP1 homomultimer is responsible for bulk proteolysis, while the homohexameric ring of the AAA+ unfoldase CLPX and the homoheptameric ring of the peptidase CLPP perform first aid in IMM-associated granular condensates, in cooperation with the AAA+ unfoldase VWA8. CLPX maximizes the flux within the heme-biosynthesis multi-enzyme metabolon, unfolding ALAS and OAT so that they can bind to their cofactor PLP, to perform transaminations at delta-carbon positions. CLPX also associates with the translation elongation factor GFM1 when a nascent peptide is misfolded. Indirectly via heme or directly via GRSF1, CLPX may also influence mtRNA-G4 processing. The heme availability also impacts the respiratory chain and many processes outside the mitochondria. Rather than performing proteolytic degradation, without assistance by the ATPase CLPX, CLPP can only trim short polypeptides from proteins and assemblies, like chymotrypsin. CLPP apparently has access to diverse phase-separated, ribonucleoprotein-containing condensates in the matrix, where the transcription, processing, translation, degradation, and extrusion of mtRNA are decided. The illustration presents the respiratory chain at each crista with its complexes I–V and in association with iron (red dots)–sulfur (yellow dots) clusters, as well as the heme quadrangular molecule. The mitoribosomal large subunit (green globe) and small subunit (light blue) are shown with the sites for aminoacyl binding (A), peptidyl extension (P), and exit to tunnel (E), where the GTPase GFM1 determines the elongation. The various terms and protein symbols are defined in the Abbreviation List below.

The recent generation of several independent *Clpp*-knockout (KO) mouse lines, by means of Gene-Trap random insertion [40] and targeted conditional technology [41], provided authentic genetic animal models of PRLTS3 with the characteristic phenotypes and allowed for the elucidation of the underlying molecular and functional deficits. Independently and in perfect agreement, the biochemical analyses of each research team showed that CLPP homo-multimer rings exist normally without CLPX association in blue-native electrophoresis, where endogenous protein complexes are resolved according to their interactive stability and molecular weight (Figure 2 in [42], Supplementary Figure EV3B in [43]). Thus, in unstressed mammalian cells, CLPP rings do not associate with the energy-providing AAA+ ATPase CLPX and cannot perform the degradation of protein substrates, which would require ATP hydrolysis [4]. Therefore, CLPP functions would normally be limited to act as a peptidase like chymotrypsin [44], trimming proteins or multi-protein assemblies rather than completely eliminating them. This concept is in agreement with a recent review where the role of CLPX and CLPP was seen in the fine-tuning of mitochondrial matrix multi-protein assemblies rather than in proteolysis [44]. CLPX as a monomer or homo-multimer ring would then employ its energy from ATP hydrolysis to unfold proteins or protein complexes without subsequent destruction. Of course, CLPX and CLPP may join forces under conditions of cell stress, e.g., when mitoribosomal translation is stalled and a misfolded nascent polypeptide has to be degraded, or after cell damage to disaggregate and cleave the toxic oligomers of ribonucleoproteins. An illustrated synopsis of this emerging scenario is provided in Figure 1, and the detailed evidence is presented in the subsequent text paragraphs with citations of recent research.

To elucidate the exact roles of CLPP serves an urgent unmet medical need, given that the modulation of CLPP activity by drugs is consistently observed to be very efficacious in counteracting solid cancers and infections [45,46,47]. Indeed, the effect of CLPP is strong even under physiological conditions: its dysfunction leads to a short stature in patients [15], and it is unclear if this represents impaired cell growth, reduced proliferation rates, or developmental deficits. In the PRLTS3 mouse models, a reduction in weight of up to 50% was observed, together with an underlying similar decrease in the nitrogen-storing amino acid L-arginine, which is consumed in the maximized biosynthesis of heme instead of fueling growth [48]. This means that nitrogen availability in L-arginine could be the limiting factor for organism growth. In a preliminary meta-analysis of CLPP substrate trapping experiments and of CLPP–null proteome profiling in many organisms from bacteria to humans, an enrichment of mitochondrial ribonucleoproteins was observed, with the unfoldase CLPX, the mitoribosomal translation factor GFM1, and the RNA degradation factor PNPT1 emerging as the proteins that most consistently interact with CLPP and show accumulation upon its loss [36]. 

## 2. Novel Evidence on CLPP and CLPX’s Functions from *Clpp*-KO Mice and PRLTS3 Patients

### 2.1. Prominent Impact of Absence of CLPP and Excess of CLPX on Mitochondrial Nucleoids

Upon the first generation of two independent *Clpp*-KO mouse embryonic stem cells by inactivating Gene-Trap insertions in intron 1 and 2 at the Texas Institute of Genomic Medicine (TIGM), the derived mice were shown to serve as authentic models of Perrault syndrome [40]. These homozygous *Clpp*-KO mice exhibited complete infertility even at an early age, an average weight reduction of up to 70% and length reduction of up to 90% from 12 weeks, impaired locomotor activity by the age of 6 months, sensorineural hearing impairment from 12 to 18 months, and a relative resistance to microbial infections [40]. In contrast to other forms of Perrault syndrome with exclusively female infertility due to primary ovarian insufficiency, CLPP absence according to mouse data also causes male infertility due to azoospermia after diplotene arrest [49]. Further analyses of the lean phenotype of several *Clpp*-KO mouse lines showed a protection from diet-induced obesity and from insulin resistance but also a deficit to adapt their body’s thermogenesis [50,51]. 

The molecular analyses of tissues showed the absence of CLPP to cause a >3-fold accumulation of the unfoldase CLPX, together with increased amounts of the mitochondrial protein chaperone mtHSP75 (but not HSPD1) [40,52]. This corresponds partially to previous observations in *Caenorhabditis elegans* studies on the unfolded protein response of mitochondria (UPRmt), where hsp-6 and hsp-60 were induced [53]. Beyond the expected impairment of proteostasis, careful quantification of mtDNA with qPCR in the testis, ovary, heart, brain, liver, and blood demonstrated a 1.5- to 4-fold increase in the mtDNA copy number [53]. This observation was not reproduced in mice with conditionally targeted *Clpp* deletion when the full-length mtDNA from the heart muscle was assessed by means of Southern blotting [43], but it was confirmed by an independent team in white adipose tissue from the Gene-Trap *Clpp*-KO mice with qPCR [51] and also in CLPP-mutant patient skin fibroblasts by means of qPCR [54]. Furthermore, the patient fibroblast analysis by means of microscopy demonstrated an enlargement in the nucleoid area, with an apparent elevation of mtDNA signals [54]. It is important to note that the increased mtDNA copy number was not accompanied by an elevated abundance of TFAM as its primary binding partner. Instead, the proteome profiling of the targeted *Clpp*-KO mouse heart tissue, Gene-Trap *Clpp*-KO mouse embryonic fibroblasts, and patient fibroblasts documented prominent accumulation of the nucleoid factor POLDIP2 [52,54,55], a protein that is known to associate with mtSSB [55] and CLPX, which maximizes the activity as well as stability of CLPXP [56]. Mechanistic analyses of the drug ZG36, which acts as a CLPP agonist, showed a converse impact, with a reduction in mtDNA to half [57]. In addition, in *Clpp*-KO testis at the early stages of spermatogenesis, a consistent accumulation was observed for the Twinky isoform of the mtDNA helicase TWNK/PEO1, which differs from the Twinkle isoform by absent binding to the D-loop [58]. This finding appears to be particularly relevant given that TWNK mutations can cause Perrault syndrome [23,59,60,61,62,63,64,65,66]. 

These observations are compatible with the concept that the absence of CLPP results in an increased dosage of mtDNA fragments rather than full-length copies, so that their assembly with associated proteins is impaired, and the generation of the polycistronic transcript may be affected. 

### 2.2. Prominent Impact of Absence of CLPP and Excess of CLPX on Mitoribosomes, Mostly on tRNA-/mRNA-Associated and rRNA-Containing mtSSU

Further evidence that CLPP and CLPX target granular components of the mitochondrial matrix was reported for mitoribosomes, initially in the targeted *Clpp*-KO mouse [43], and then in the Gene-Trap *Clpp*-KO mouse as well [42]. 

Regarding ribosomal RNA, the 12S rRNA and MRPS15-MRPS35 protein components of the mitoribosomal 28S small subunit (mtSSU) showed a much more elevated abundance than the 16S rRNA and MRPL12-MRPL37 protein components of the large subunit of mitoribosomes (mtLSU) in *Clpp*-KO mice [41]. Further analyses of the co-migration of mitochondrial proteins in blue-native gel electrophoresis to define their interaction in assembled complexes confirmed a general accumulation of all the components of the mtSSU in a *Clpp*-KO testis, brain, and heart in the absence of CLPX co-migration [42]. 

Regarding the translation-associated enzymes, the elongation factor GFM1 (also known as EFG1, an ortholog of bacterial *fusA*-encoded EF-G, see [67]) exhibited an elevated protein abundance in *Clpp*-KO mice as well, along with abnormal sedimentation in sucrose gradients [41]. Indeed, the co-accumulation of CLPX together with its interactor GFM1 was subsequently confirmed in proteome profiling studies of mouse brains, MEFs, and patient fibroblasts [54]. This is in agreement with the notion that CLPX not only acts in heme biosynthesis but is also able to target the GFM1-associated L7/L12 stalk and central protuberance of mitoribosomal LSU to act in the translation elongation/recycling apparatus [68,69,70,71,72].

These findings identify the molecular details that underlie previous observations that CLPXP is necessary to rescue stalled translation complexes by unfolding the mitoribosome, so that translation elongation via the addition of a CAT tail to the nascent misfolded polypeptide can occur. CLPXP then eliminates this aberrant translation product before its aggregation tendency has toxic effects [73,74,75,76]. 

### 2.3. Prominent Impact of Absence of CLPP and Excess of CLPX on Mitochondrial RNA Processing Granules

A third line of evidence on the role of CLPP in mitochondrial matrix granules concerns the RNA processing compartment. It was observed that mtSSU rRNA accumulates in *Clpp*-KO mice [41]. ERAL1 serves as a mitochondrial rRNA chaperone, while the 12S rRNA associates with ribonucleoproteins to form the mtSSU. Indeed, ERAL1 exhibited not only an elevated protein abundance in *Clpp*-KO heart mitochondria but also abnormal sedimentation in sucrose gradients [41]. Again, ERAL1 accumulation appears to be particularly relevant, given that ERAL1 mutations can trigger Perrault syndrome [25,77,78,79]. 

*Clpp*-KO-triggered accumulation was also documented for a few mitochondrial tRNAs [41]. In particular, the tRNAs for valine (Val) and phenylalanine (Phe) exhibited higher aminoacylation in a *Clpp*-KO heart [41]. Therefore, it is interesting that a very selective protein accumulation exists for mtLSU components like MRPL18 and MRPL38, which assemble with tRNA-Val/Phe in the central protuberance of mitoribosomes, and that this CLPP-null effect on the central protuberance subunits of the mtLSU is conserved across eukaryotes until the ascomycete fungus *P. anserina* [42,48]. This selective impact on the mtLSU may also be relevant for mtDNA and the cytosolic stress response: MRPL38 influences the maintenance of the mitochondrial nucleoid, at least in yeast [80]. Furthermore, there is a cytosolic isoform of MRPL18, which modulates the ribosomal translation of molecular chaperones after cell stress [81] and can thus influence the UPR outside of mitochondria. 

A key role of a tRNA-associated pathology in CLPP-dependent pathogenesis is also evident from human genetics data. Mutations in the mitochondrial tRNA–aminotransferases for histidine and leucine, HARS2 and LARS2, cause the typical features of Perrault syndrome [21,30,82,83,84,85,86], while mutations in the mitochondrially encoded tRNA sequences trigger different and mostly neurodegenerative phenotypes, including progressive deafness [87]. Mirroring a joint pathogenetic pathway for different variants of Perrault syndrome, *Clpp*-KO testes from the early stages of spermatogenesis contain elevated amounts of HARS2 [42]. It is furthermore worth noting that the deleterious effects of mutations in DARS2 (mitochondrial tRNA–aspartate aminotransferase) in mice can be partially rescued by an absence of CLPP [41,88], so in conditions of enhanced mitochondrial RNA processing and translation blockade, it can be advantageous to have a CLPP loss-of-function that reduces UPRmt and prolongs the lifespan.

The folding of mitochondrial tRNAs and rRNAs represents another pathway that is affected both by the impact of CLXP on heme and by the impact of CLPP on ribonucleoprotein condensates. Bacterial tRNA and rRNA contain guanine-rich sequences that can adopt quadruplex structures [89]. Also, for mammalian cytosolic ribosomes, the importance of such rRNA quadruplexes for mature conformation has already been documented [90]. When guanine-rich sequences adopt a quadruplex conformation (G-tetrads) with four RNA or DNA strands (RNA-G4 or DNA-G4), their structure can be stabilized by an association with quadrangular porphyrin and heme molecules [90,91,92,93]. This interaction may activate peroxidase- or oxidase-mimicking features in this DNAzyme/RNAzyme complex [92,93], may modify the compaction and processing of DNA/RNA [94], and is crucial in ribosomes for optimal translation efficiency [95]. The high abundance of such rRNA-G4 structures even limits the bioavailability of heme in cells [90]. This pathway seems to be altered in PRLTS3, in view of the selective accumulation of the RNA granule factor GRSF1 (G-Rich Sequence Factor 1) in *Clpp*-KO tissues [41,54]. Within mitochondria, GRSF1 is responsible for non-coding RNA in the G4 conformation [96,97]. GRSF1 interacts with RNase P to influence the cleavage of polycistronic transcripts [98], and its dysfunction leads to RNA processing defects, the accumulation of mtRNA breakdown products, as well as increased levels of dsRNA and RNA:DNA hybrids [99]. These problems lead to the formation of distinct mitochondrial dsRNA foci [100]. In addition, GRSF1 dysfunction triggers the abnormal loading of mRNAs and lncRNAs on the mitochondrial ribosome and impaired ribosome assembly [101]. GRSF1 also influences the degradation of mtRNA in the degradosome in cooperation with PNPT1 [96,102]. Overall, it is not surprising that GRSF1 is also involved in iron toxicity like CLPX [103] and in lean body phenotypes like CLPP [104,105]. GRSF1 was observed in protein–protein interactions with CLPX [36].

RNA-G4 structures also control the activity of the mitochondrial GTPase NOA1 (also known as C4ORF14) for mitoribosomal assembly [106,107,108]. NOA1 was also identified as a CLPXP target protein [109]. 

The joint roles of absent CLPP and excess CLPX during the assembly of mitoribosomes are further supported by the selective accumulation of VWA8 in *Clpp*-KO tissues [42]. The mitochondrial matrix protein VWA8 [110] contains a domain that is related to porphyrin chelatases [42], so it might interact with heme or its precursors. VWA8 also contains an AAA+ unfoldase domain, whose protein targets are undefined in mammals. Its yeast ortholog midasin (also known as Rea1) was clearly shown to be responsible for the maturation of the mitoribosomal LSU [111,112,113]. 

With excess heme being released from mitochondria in PRLTS3, abnormal G-tetrad processing might also occur in the nucleus, where homologous recombination is known to depend on DNA-G4 structures [114]. Thus, the complete infertility of PRLTS3 patients, with the abortion of nuclear meiosis-I after diplotene arrest [49], might partially be a consequence of defective G4 conformations. 

Furthermore, the mitochondrial RNA granule factor LRPPRC undergoes selective accumulation in *Clpp*-KO tissues [41,54]. LRPPRC is known to modulate the poly(A) tail of mRNAs in mitochondria [115,116,117,118,119], and its dysfunction influences the efficiency of the RNA degradosome together with the accumulation of toxic dsRNA [120].

Jointly, all this evidence indicates that the processing of polycistronic mtRNA, which is transcribed from mtDNA and then cleaved to tRNAs, rRNAs, other non-coding RNAs, and mRNAs, is selectively altered by the absence of CLPP. CLPP could trim components that are stuck within the RNA–protein complexes. CLPX clearly has a function in the disassembly of stalled translation complexes and might play a role in the G4 conformation of rRNA, which is important for the assembly of mitoribosomes.

### 2.4. Prominent Impact of Absence of CLPP and Excess of CLPX on Mitochondrial D-Foci Where RNA Degradation, Extrusion, and Innate Immunity Activation Are Decided

A fourth indication of the role of CLPP for mitochondrial matrix granules concerns the RNA degradosome in the so-called D-foci [121,122,123]. Its main component, the ribonuclease PNPT1 (which is orthologous to the bacterial polynucleotide phosphorylase/polyadenylase pnp, or PNPase), is associated with CLPP and is dysregulated upon CLPP deletion, exhibiting consistency in hosts ranging from *Escherichia coli* to mice [36]. Together with the RNA helicase SUPV3L1 (best known as SUV3, see [124]) and the RNA-G4-quadruplex modulating factor GRSF1, PNPT1 eliminates dsRNA, acting as 3′-5′ exonuclease [102,122,125,126,127,128,129], and even its bacterial ortholog pnp is responsible for antiviral immunity [130]. The matrix degradosome in D-foci appears to act not only on the abundant mtRNA, since PNPase and SUV3 show a preference for mtDNA [131,132,133,134,135]. Similarly to mutations in CLPP, mutations in PNPT1 are also the cause of progressive deafness and of a sensory neuropathy with ataxia [136,137,138,139,140,141]. PNPT1 dysfunction causes the accumulation of toxic dsRNAs and their extrusion from mitochondria into the cytosol, where antiviral innate immunity responses are activated [142]. Again, the homozygous absence of CLPP or heterozygous absence of mtDNA-binding TFAM in the mitochondrial matrix triggers cytosolic antiviral innate immunity responses, like the induction of the AAA+ unfoldase RNF213. This unfoldase is also activated by toxic dsRNA mimics such as poly(I:C) administration [143]. In *Clpp*-KO mouse brains and MEFs, the selective activation of various cytosolic sensors for toxic DNA and RNA was documented [144]. The problems in the packaging of mtDNA and in degrading/extruding toxic nucleic acids from mitochondria in *Clpp*-KO cells were shown to activate antiviral cytosolic responses via the cGAS/STING pathway [145]. 

Overall, the resulting steady-state activation of type I interferon signaling explains the marked resistance of CLPP-null mice to bacterial and RNA/DNA virus infections [40,145].

As a preliminary conclusion, the above four paragraphs represent solid evidence that CLPP has a selective impact on matrix granules in which RNA is a component, which mediates the liquid–liquid phase separation (LLPS) around these condensates.

### 2.5. Prominent Impact of Absence of CLPP via CLPX Accumulation on Heme Biosynthesis and Incorporation into Complex-IV of the Respiratory Chain

The absence of CLPP causes a several-fold accumulation of CLPX, as explained above. CLPX has an important role in the heme metabolism multi-enzyme complex, which is associated with the IMM [146], and serves to separate ferrous iron and reduced porphyrin intermediates from unwanted reactions in the matrix [147,148]. This multi-enzyme chain was previously shown to serve as a metabolon, which is by definition held together by non-covalent interactions, as protein condensate with minimal hydration, to allow for substrate channeling and maximal productivity [149,150,151]. The complex contains ALAS, which is the first enzyme of heme biosynthesis and whose product delta-aminolevulinic acid (deltaALA) is exported from mitochondria into the cytosol. It also contains CPOX-PPOX-FECH on different IMM surfaces as the three terminal enzymes of the biosynthesis chain, whose product, heme, is incorporated into complexes II, III, and IV of the respiratory chain within the IMM [147,152]. ALAS is furthermore associated with SUCLA2 in differentiating erythroid cells [147]. This IMM-associated multi-enzyme complex also serves as a bridge [153,154] between at least three transmembrane proteins. Firstly, MFRN1 (also known as SLC25A37 or mitoferrin-1), which imports iron into the mitochondrial matrix [155]; secondly, ABCB10, which exports biliverdin to the cytosol [156]; and thirdly, ABCB7, which exports glutathione-coordinated iron–sulfur clusters to the cytosol [157], are connected to the IMM-associated heme biosynthesis complex, according to several consistent reports. There is still debate [147] about whether the tight association of IMM transmembrane proteins with this metabolon goes beyond the biliverdin/zinc-mesoporphyrin transporter ABCB10 [158,159,160] to include also the TMEM14C protoporphyrin-IX transporter [161,162,163,164], the protoporphyrin-IX transport modulator ANT2 [165], and the glutathione/succinate transport modulator OGC [166,167]. Enzyme complexes with similar isolations of reaction intermediates from the surrounding matrix have also been observed, e.g., during L-arginine metabolism and bacterial cobalamin metabolism [168,169]. Heme and porphyrins are compounds that need a hydrophobic environment [170,171,172,173]. According to recent human genetics findings, mutations in FECH, ALAS2, and CLPX [174] underlie most cases of the disorder erythropoietic protoporphyria, while mutations in ALAS2 and in the mitochondrial glycine transporter SLC25A38 are the most frequent causes of congenital sideroblastic anemia [175].

CLPX was shown to unfold ALAS, so that its cofactor pyridoxal-5′-phosphate (PLP) can bind and activate it to consume succinate-CoA and glycine for the production of the heme precursor delta-aminolevulinic acid (deltaALA) with optimal efficiency [5,37,38,176,177,178]. CLPXP was claimed to be responsible for ALAS degradation [179]. In the *Clpp*-KO mouse, the consequent elevation of CLPX abundance will also unfold OAT (ornithine delta-aminotransferase), so that PLP binds to it and triggers the consumption of L-arginine and L-ornithine via a delta-transaminase and delta-aminomutase reaction to produce GSA as a precursor of heme. In parallel, a recruitment of L-glutamate occurs into maximized deltaALA generation via the accumulation of the enzyme ALDH18A1 (also known as delta-1-pyrroline-5-carboxylate synthase) [48,52]. Thus, both ALAS and OAT acquire the ability to perform transaminations at the delta-carbon position, when CLPX unfolds them and enables them to bind to PLP as cofactor [180]. At the same time, iron accumulates in *Clpp*-KO tissue, together with the heavy metals molybdenum, cobalt, and manganese [42]. Thus, heavy metal toxicity and ferroptosis [181,182] may also be part of PRLTS3 pathogenesis mechanisms. The accumulation of the metal- and heme-binding protein COX15 and the preferential affection of respiratory complex-IV in the IMM of *Clpp*-KO mice can thus be explained as a consequence of iron/heme dysregulation [42]. Indeed, the expression dysregulation of the heme-binding, mtDNA-encoded *Cox1* membrane subunit in complex-IV stood out across the *Clpp*-KO testis, heart, liver, and brain as the main molecular underpinning of respiratory dysfunction [40]. While heme is a protoporphyrin-IX that is chelated with Fe^2+^, plant chlorophyll is a protoporphyrin chelated with Mg^2+^, so both heme and chlorophyll biosynthesis depend on ALAS control by PLP and CLPX. Indeed, the regulation of heme/chlorophyll metabolism by CLPX is conserved from bacteria across phylogenesis to plants [183,184,185,186,187]. 

Unsurprisingly, the accumulation of CLPX in *Clpp*-KO tissues not only modulates the binding of PLP to target enzymes but also leads to increased amounts of the PLP storage/transport protein PLPBP in some cell types [42]. 

Altogether, CLPX appears to fine-tune the biosynthesis and maturation of porphyrins and heme, with marked consequences for iron and heavy metal utilization, as well as respiratory competence, through the continuous modulation of the IMM-associated multi-enzyme complex, which channels hydrophobic reaction intermediates and isolates them from the aqueous phase. 

### 2.6. Prominent Impact of Absence of CLPP on Fe-S Cluster Containing Peripheral Arm of Respiratory Complex-I

The fine-tuning of multi-protein assemblies, rather than proteolytic degradation, also seems to characterize the selective role of the absence of CLPP and accumulation of CLPX for the respiratory complex-I N-module [188]. Complex-I consists of a membrane arm, embedded in the lipid bilayer of the IMM, and a peripheral arm with the N/Q modules that protrudes into the aqueous phase of the matrix. The two modules serve to surround, isolate, and channel electrons into a tunnel within the IMM and to protect the many embedded iron–sulfur (Fe-S) clusters from oxidation [189,190,191,192,193]. The absence of CLPP only results in a mild reduction in complex-I dependent state 3 respiration in mouse heart mitochondria but not in other tissues, so the mutation-triggered functional deficit is subtle [40,43]. As a molecular underpinning, it was clearly shown that the turnover of the core subunit NDUFV1-NDUFV2-NDUFS2 in the NADH-oxidizing N-module of complex-I has a selective dependence on CLPP in an ongoing exchange process where oxidatively damaged, inactive N-modules are substituted on the tip of the complex-I peripheral arm [194]. In addition, the selective accumulation of SFXN4 in *Clpp*-KO tissue, as a component of the complex-I assembly machinery that controls metal association, indicates that the biogenesis of complex-I may be altered [42]. It has to be mentioned, however, that none of the established complex-I subunits accumulate in *Clpp*-KO mouse tissues and that proteome profiling in the CLPP-null fungal eukaryote *P. anserina* did reveal some accumulated complex-I subunits but not for any specific module [48]. Thus, we assume that complex-I assembly is not a primary and conserved target of CLPP. Our team has observed upregulations of most factors in the iron–sulfur cluster (ISC) biogenesis pathway, most strongly for the 4Fe–4S cluster generating enzymes, in the brain of *Clpp*-KO mice, but of course, this effect may simply represent a molecular adaptation to maintain sufficient ISC production despite the maximized iron utilization for heme biosynthesis.

Clearly, the functions of CLPP and CLPX seem to consist of the rapid refolding/trimming or substitution of a selected subunit within a complex that keeps working, but not the complete disassembly and disposal of entire respiratory complexes or supercomplexes.

## 3. Phase-Separated Condensates in Mitochondria and the Cytosol

Research over the past four years showed that nucleoid components, the RNA processing granules, and the RNA degradasome of bacteria and mitochondria assemble in phase separation [100,195,196,197,198,199]. The original concept of LLPS over twenty years ago [200,201] was derived from lipid droplets, where components can move freely within a round compartment that excludes the aqueous phase. In the meantime, it has become clear that such condensates do not need to be liquid but can also assume a gelatinous or even solidified state, particularly in a disease context [202]. Although the mobility of individual components within the condensate may be high, they would certainly move along given structures within the phase-separated condensate, e.g., in the case of nucleolar ribosome biogenesis, mtDNA transcription, or the processing of polycistronic mtRNA. Thus, these condensates may be defined by the multivalent interaction forces that keep long and flexible molecules such as lipids or RNA together [203,204]. They are also defined by the vulnerable reaction intermediates that need protection from the aqueous or membrane phases, such as cleaved unfolded RNA without modifications, unchelated porphyrins that are unassembled with proteins, or pre-fibril oligomers with a propensity to disrupt membranes [205,206,207]. Regarding its multivalent interaction forces and its long flexible structure, RNA was the prime example for understanding LLPS, based on the phase contrast during the microscopic visualization of the nucleolus. Therefore, other RNA-containing granules in the nucleus and cytosol (e.g., paraspeckles, Cajal bodies, U bodies, PcG bodies, Balbiani bodies, stress granules, P-bodies, germ granules, and RNA transport granules) constituted an early focus of LLPS research (Figure 1 in [202]). It was shown that the ribonucleoproteins also contribute to phase separation, with some binding domains deciding the specificity of interactions (known as “stickers”), while the intrinsically disordered regions (IDRs) that often intervene have solvation properties that influence the density transition (known as “spacers”) [203]. Indeed, it was proposed that cells use RNAs and IDR proteins to separate multi-enzyme complexes such as glycolysis into granular compartments that have a different phase than the surrounding cytosol [208]. Other mitochondrial metabolons, such as the TCA cycle, heme biosynthesis, urea cycle, respiratory chain, and breakdown of branched amino acids, also require the efficient channeling of reaction intermediates, which are usually achieved by tight subunit docking and by hydrophobic interactions [209]. Thus, these metabolons might also be separated from the aqueous phase by the multivalent forces of associated non-coding RNA. 

In summary, the recently defined targets of CLPP and CLPX are all condensates where phase separation or multi-enzyme assembly protect unstable reaction intermediates from the aqueous phase, and which frequently need a rapid repair of individual subunits while these assemblies keep fulfilling their function. It seems plausible that CLPP and CLPX have the ability to access these condensates and provide the necessary first aid. 

## 4. Proposal

Altogether, it may be impossible to define consistent protein targets of CLPX and CLPP across phylogenesis, given that each organism is adapted to a different environment, has specific metabolic needs, and has to protect other reaction intermediates inside phase-separated multivalent or hydrophobic condensates from the aqueous phase. The polypeptide sequences of degrons that are recognized by CLPX in *E. coli* might differ from such sequences in mice, and the cleavage pattern of CLPP, while it fine-tunes multi-enzyme complexes, may vary according to steric constraints. There could be no protein that is exclusively the substrate of CLPXP-mediated proteolysis, and all matrix proteins might be finally degraded by LONP1. This is exemplified by CLPX, which is certainly a prominent example of a protein whose abundance depends on CLPP in all organisms studied [36], and yet, its proteolytic destruction was observed to be executed by LONP1 [210]. Instead of eliminating specific proteins in the mitochondrial matrix, CLPX and CLPP seem to have unique access properties to granular compartments, where they can rescue a suboptimal or stalled process, either by unfolding a protein or by the excision of a misassembled component, so that the multi-enzyme complex can improve its performance (see Figure 1). Overall, it is necessary to validate whether the *Clpp*-KO mouse evidence holds true in other organisms, i.e., that the specific roles of CLPX and CLPP are defined by the granular compartments that they are monitoring. This would be analogous to most other peptidases in mitochondria, where MPP cleaves all precursor proteins at the import pore, m-AAA and i-AAA are responsible for protein quality control at either IMM face, PARL cleaves proteins within the IMM, and OMA1/HTRA2 performs surveillance in the intermembrane space [211,212,213,214]. Our proteome identification of specific factors whose abundance depends on CLPP will also be useful (more so than unspecific mitochondrial–respiratory assays) for comparing the efficacy of drugs that activate or inhibit CLPP. This research area is rapidly advancing and holds great promise for the treatment of cancer and infections. While the authors are no experts in this field, we recommend several innovative reviews and articles produced over the past 10 years on CLPP-modulating drug compounds [215,216,217,218,219,220,221,222,223,224,225,226,227,228,229,230,231,232,233] and relevant structure/binding studies [45,234,235,236,237,238,239,240,241,242,243,244] for further reading. A better understanding of CLPXP-dependent UPRmt will also help clarify how extra-mitochondrial signals (such as extruded mt-dsRNA, perhaps mtRNA-G4, and associated ribonucleoproteins) trigger responses of the nucleus and the endoplasmic reticulum UPR, a basic research field where mechanisms are investigated in yeast and nematodes but are poorly defined in mammals at present [41,245,246,247,248,249,250].

## Data Availability

No new data were created or analyzed in this study. Data sharing is not applicable to this article.

## Abbreviations

4Fe–4S	iron–sulfur clusters consisting of two interleaved 4Fe- and 4S-tetrahedra
AAA+	ATPases Associated with diverse cellular Activities and other ring-shaped P-loop NTPases
ABCB7	ATP Binding Cassette Subfamily B Member 7
ABCB10	ATP Binding Cassette Subfamily B Member 10
ALAS	delta-Amino-Levulinic Acid Synthase
ALAS2	delta-Amino-Levulinic Acid Synthase 2, erythroid-specific
ALDH18A1	Aldehyde Dehydrogenase 18 family member A1, =P5CS, delta-1-Pyrroline-5-Carboxylate Synthase
ANT2	Adenine Nucleotide Translocator 2, =SLC25A25
ATP	Adenosine Tri-Phosphate
ATPase	Adenosine Tri-Phosphatase
CAT-tail	C-terminal alanine and threonine tail
cGAS	cyclic GMP-AMP Synthase
CLPP	Caseinolytic Mitochondrial Matrix Peptidase Proteolytic Subunit
CLPX	Caseinolytic Mitochondrial Matrix Peptidase Chaperone Subunit X
CODAS	multiple anomalies syndrome with Cerebral, Ocular, Dental, Auricular and Skeletal anomalies
*Cox1*	mitochondrially encoded Cytochrome C Oxidase I, mRNA
COX15	Cytochrome C Oxidase assembly homolog COX15
CPOX	Copro-Porphyrinogen OXidase
D-foci	degradosome granules in mitochondrial matrix
D-loop	displacement loop within the mtDNA
DARS2	Aspartyl-tRNA Synthetase 2, mitochondrial
deltaALA	delta-aminolevulinic acid
DNA	Deoxyribo-Nucleic Acid
DNAzyme	catalytically active DNA sequences
dsRNA	double-stranded RNA
EF-G	Elongation Factor G
EFG1	G Elongation Factor, mitochondrial 1
ERAL1	Era (*E. coli*) -Like 12S mitochondrial rRNA chaperone 1
Fe^2+^	divalent cation of iron, ferrous iron
FECH	Ferrochelatase
Fe-S	iron–sulfur
G4	Guanine quadruplex where RNA or DNA acquires four-stranded conformation
GFM1	G elongation Factor Mitochondrial 1
GRSF1	G-rich RNA Sequence-binding Factor 1
GSA	Glutamate-5-Semi-Aldehyde
GTP	Guanosine-5′-triphosphate
HARS2	Histidyl-tRNA Synthetase 2, mitochondrial
hsp-6	heat shock protein family B (small) member 6
hsp-60	heat shock protein family D (hsp60) member 1, =human HSPD1
HSPD1	heat shock protein family D (hsp60) member 1, =human HSP60, chaperonin
HTRA2	High-Temperature-Requirement A serine peptidase 2
i-AAA	ATP-dependent zinc metalloprotease YME1 (*S. cerevisiae*) -Like 1
IDR	Intrinsically Disordered Region
IMM	Inner Membrane of Mitochondria
ISC	iron–sulfur cluster
KO	knockout
LARS2	Leucyl-tRNA Synthetase 2, mitochondrial
lncRNA	long non-coding RNA
LLPS	liquid–liquid phase separation
LONP1	Lon Peptidase 1, Mitochondrial
LRPPRC	Leucine-Rich Pentatrico-Peptide Repeat Containing
m-AAA	AFG3-like matrix AAA peptidase subunit 2 and SPG7 matrix AAA peptidase subunit paraplegin
MEFs	mouse embryonic fibroblasts
MFRN1	mitoferrin 1, =SLC25A37, Solute Carrier Family 25 Member 37
Mg^2+^	divalent cation of magnesium
MPP	mitochondrial processing peptidase
MRPL12	Mitochondrial Ribosomal Protein L12
MRPL18	Mitochondrial Ribosomal Protein L18
MRPL37	Mitochondrial Ribosomal Protein L37
MRPL38	Mitochondrial Ribosomal Protein L38
MRPS15	Mitochondrial Ribosomal Protein S15
MRPS35	Mitochondrial Ribosomal Protein S35
mtDNA	mitochondrial DNA
mtHSP75	mitochondrial heat shock protein 75, =human TRAP1
mtLSU	mitoribosomal 39S large subunit
mtRNA	mitochondrial RNA
mtSSB	mitochondrial single-stranded DNA binding protein
mtSSU	mitoribosomal 28S small subunit
mt-tRNA	mitochondrial transfer RNA
NADH	Nicotinamide Adenine Dinucleotide, reduced form
NDUFS2	NADH:Ubiquinone Oxidoreductase Core Subunit S2
NDUFV1	NADH:Ubiquinone Oxidoreductase Core Subunit V1
NDUFV2	NADH:Ubiquinone Oxidoreductase Core Subunit V2
NOA1	Nitric Oxide Associated 1
NTPase	Nucleoside-Tri-Phosphatase
OAT	Ornithine delta-Amino-Transferase
OGC	2-Oxoglutarate/Malate Carrier protein, mitochondrial
OMA1	Overlapping with the M-AAA protease 1 homolog, zinc metallopeptidase
OMM	Outer Membrane of Mitochondria
PARL	Presenilin-Associated Rhomboid-Like
PcG bodies	polycomb bodies
PEO1	Progressive External Ophthalmoplegia 1 protein = TWNK
Phe	phenylalanine
PLP	Pyridoxal-5′-Phosphate
PLPBP	PLP-binding protein
PNPase	Polyribo-Nucleotide Phosphorylase/Nucleotidyl-Transferase 1 = PNPT1 in human
PNPT1	Polyribo-Nucleotide Phosphorylase/Nucleotidyl-Transferase 1 = PNPase
POLDIP2	DNA Polymerase Delta Interacting Protein 2
poly(A) tail	poly(adenine) tail of messenger RNAs
poly(I:C)	poly(inosinic:cytidylic) acid
PPOX	Proto-Porphyrinogen OXidase
PRLTS3	Perrault Syndrome type 3
PRORP	Protein-Only RNase P catalytic subunit
qPCR	quantitative Polymerase Chain Reaction
RMND1	Required for Meiotic Nuclear Division 1 homolog
RNA	Ribo-Nucleic Acid
RNA-G4	RNA, guanine-rich, in quadruplex conformation
RNase	ribonuclease
RNAzyme	catalytically active RNA sequences
RNF213	Ring Finger protein 213
rRNA	ribosomal RNA
SFXN4	Sideroflexin 4
SLC25A37	Solute Carrier family 25 member 37, Mitoferrin 1
SLC25A38	Solute Carrier Family 25 Member 38, mitochondrial glycine transporter
STING	STimulator of INterferon response cGAMP interactor 1
SUCLA2	Succinate–CoA Ligase ADP-forming subunit β
SUPV3L1	SUV3, Suv3-like RNA helicase
TCA	Tri-Carboxylic Acid cycle, =Krebs cycle
TFAM	Transcription Factor A, Mitochondrial
TIGM	Texas Institute of Genomic Medicine
TMEM14C	transmembrane protein 14C
tRNA	transfer RNA
TWNK	Twinkle mtDNA helicase, =PEO1
UPR	unfolded protein response
UPRmt	unfolded protein response in mitochondria
Val	valine
VWA8	von Willebrand Factor A domain containing 8
